# Supplementary material for: Comparative multiomics analysis of cell physiological state after culture in a basket bioreactor
Source: Sci Rep. 2022 Nov 23;12:20161. doi: 10.1038/s41598-022-24687-4 (PMC9686226; doi:10.1038/s41598-022-24687-4)
Supplement: Supplementary file 1 — Supplementary Information 1. [file 41598_2022_24687_MOESM1_ESM.zip › raw data/Transcriptomic raw data/7.AS/2.dasplot/TE1vsTC1/RI/17_BAIAP3_NW_023666068.1_29846619_29847005_-@NW_023666068.1_29845977_29847005_-@NW_023666068.1_29845977_29846161_-.pdf]

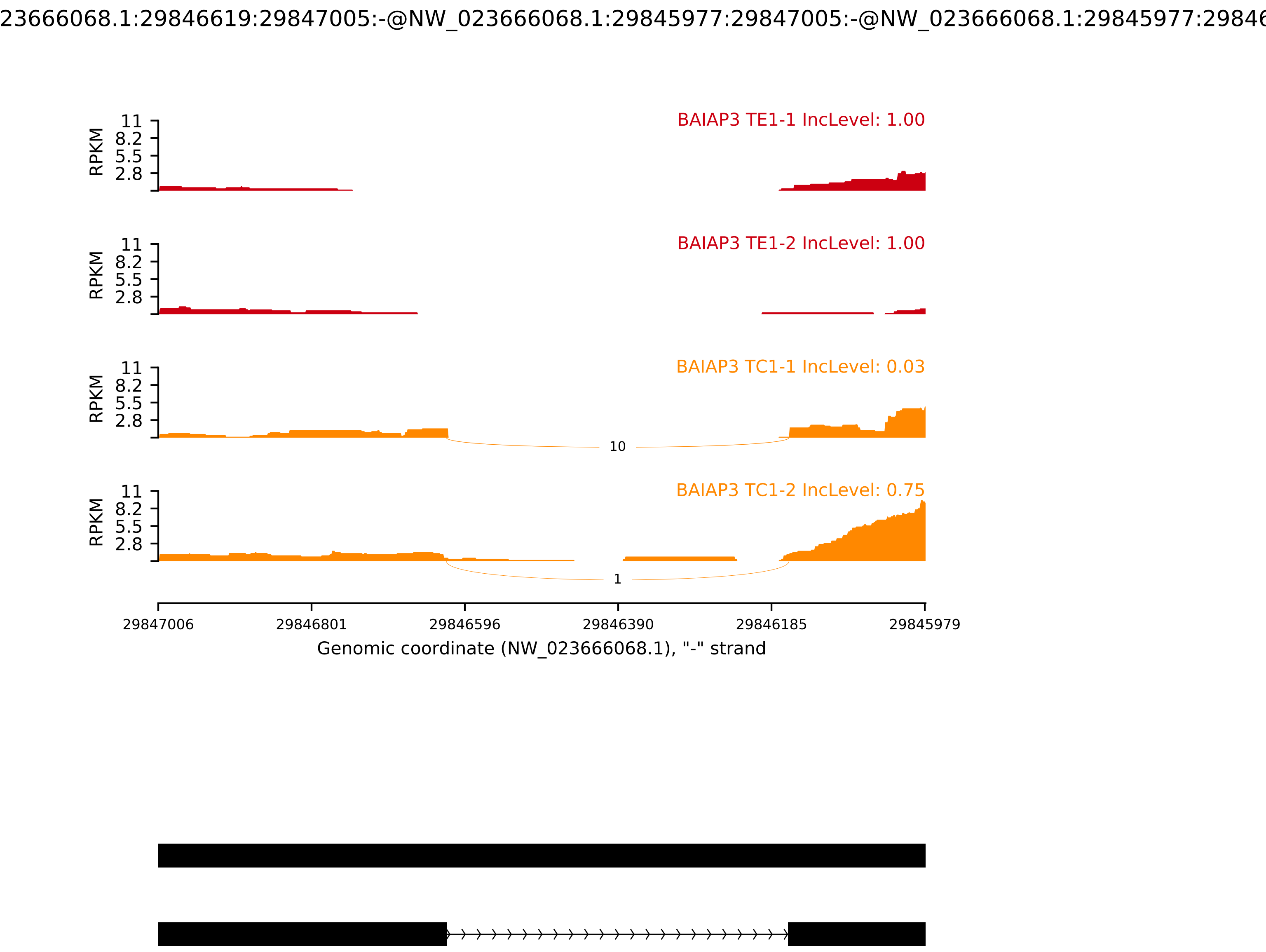

23666068.1:29846619:29847005:-@NW_023666068.1:29845977:29847005:-@NW_023666068.1:29845977:2984
BAIAP3 TE1-1 IncLevel: 1.00
BAIAP3 TE1-2 IncLevel: 1.00
BAIAP3 TC1-1 IncLevel: 0.03
BAIAP3 TC1-2 IncLevel: 0.75
RPKM
11
8.2
5.5
2.8
10
1
29847006
29846801
29846596
29846390
29846185
29845979
Genomic coordinate (NW_023666068.1), "-" strand
